# Delivering a Group-Mediated Weight Loss and Activity Program to Older Adults With Chronic Pain in the MORPH Study

**DOI:** 10.1093/geroni/igab046.1089

**Published:** 2021-12-17

**Authors:** Jason Fanning, Amber Brooks, Barbara Nicklas, W Jack Rejeski

**Affiliations:** 1 Wake Forest University, Winston Salem, North Carolina, United States; 2 Department of Anesthesiology, Wake Forest School of Medicine, North Carolina, United States; 3 Wake Forest School of Medicine, Winston-Salem, North Carolina, United States; 4 Department of Health and Exercise Science, Wake Forest University, North Carolina, United States

## Abstract

Chronic pain in aging is a potent cause and consequence of obesity, inactivity, and prolonged sedentary behavior, making these especially important targets for behavioral intervention. This study aimed to refine a theory-based group-mediated diet and sedentary behavior intervention for older adults with chronic pain. Participants (N=28) attended 12 weekly group meetings generally in home via WebEx and used an mHealth self-monitoring app as they attempted to move more often and reduce caloric intake. Relative to a control condition, the program produced improvements in physical function (η^2=.08), pain intensity (η^2=.12), sedentary time (η^2=.07), and weight loss (η^2=.21). Key findings related to effective remote group intervention delivery included: (1) the importance of a self-efficacy-enhancing technology orientation; (2) the value of small group bonding activities to seed communication; and (3) the impact of software choice on interpersonal communication. We will discuss the value of these findings for future remote intervention design.

